# Supplementary figures and images for: Investigating the lignocellulolytic gut microbiome of huhu grubs (Prionoplus reticularis) using defined diets and dietary switch
Source: PeerJ. 2024 Jul 2;12:e17597. doi: 10.7717/peerj.17597 (PMC11225714; doi:10.7717/peerj.17597)

**A**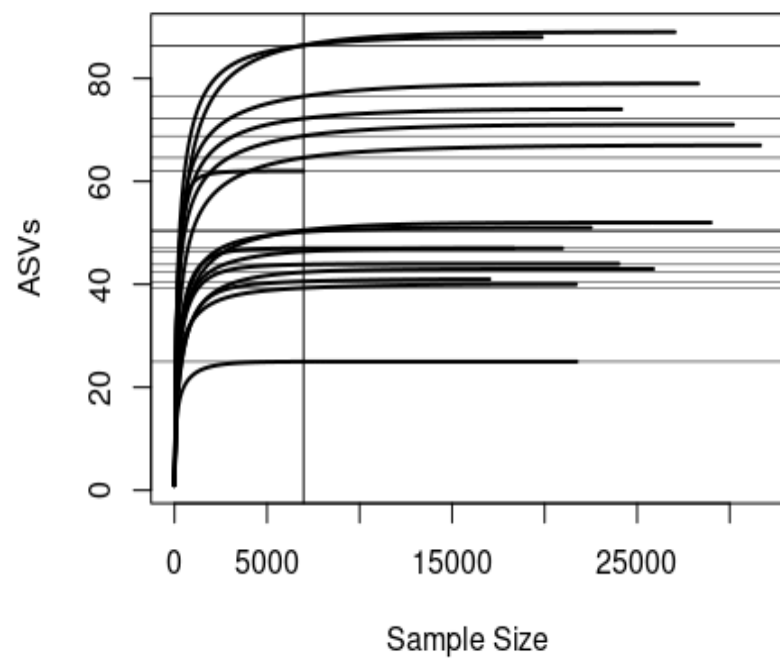**B**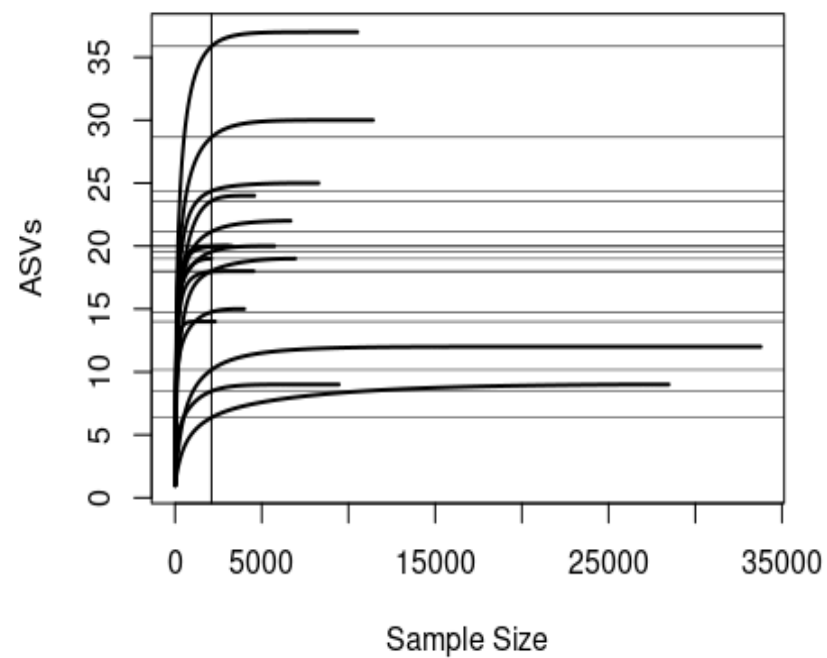

Supplement: Supplemental Information 1 — Rarefaction was performed using the raw ASVs data subsampling 10,000 times. The size of the subsample used for rarefying corresponds to the smallest library, in each data set, and is shown as a vertical line. The rarefied species richness is shown as horizontal lines. [file peerj-12-17597-s001.pdf]

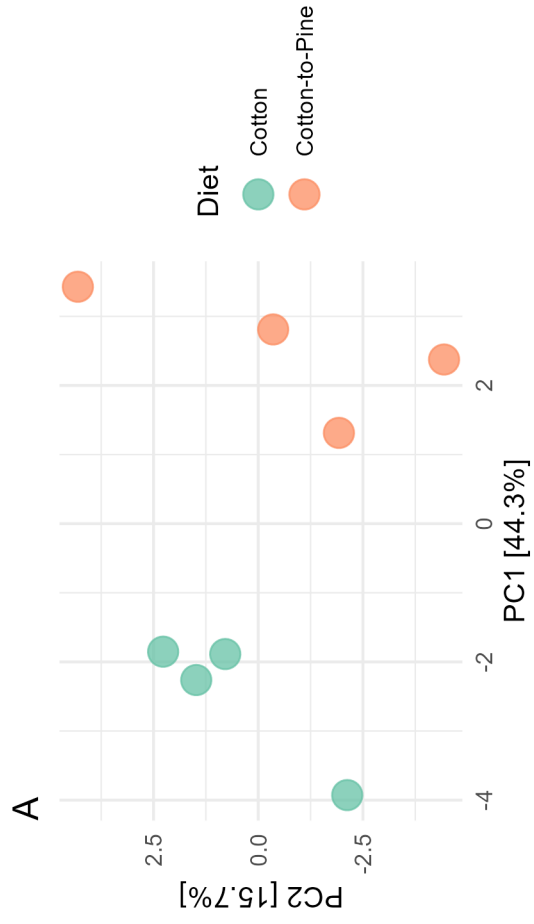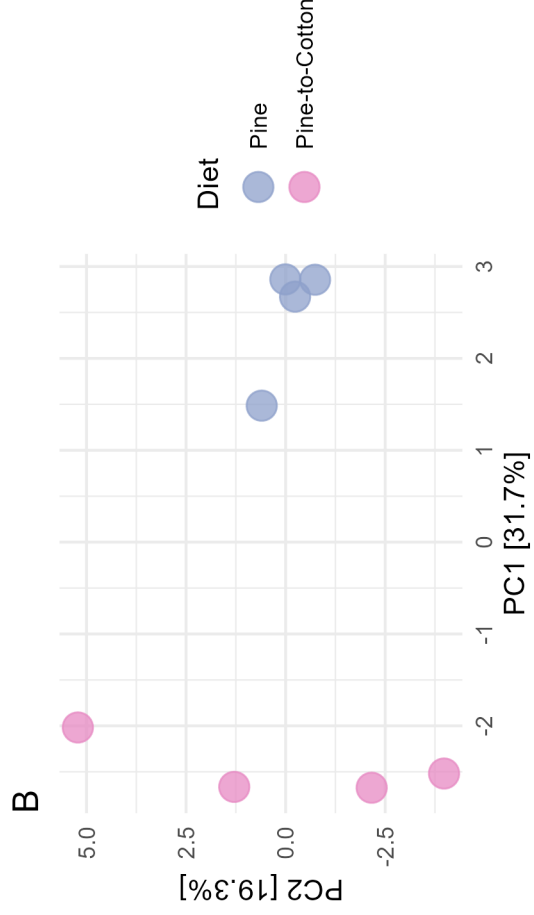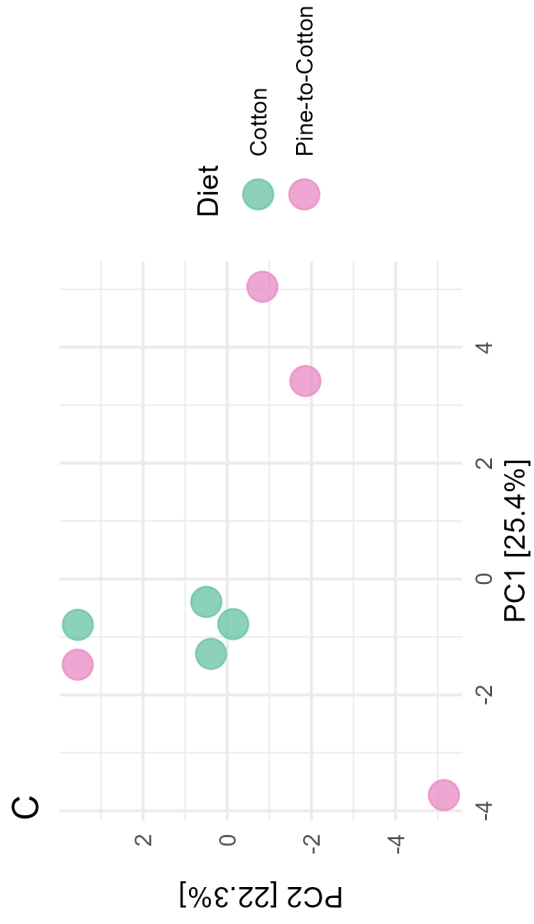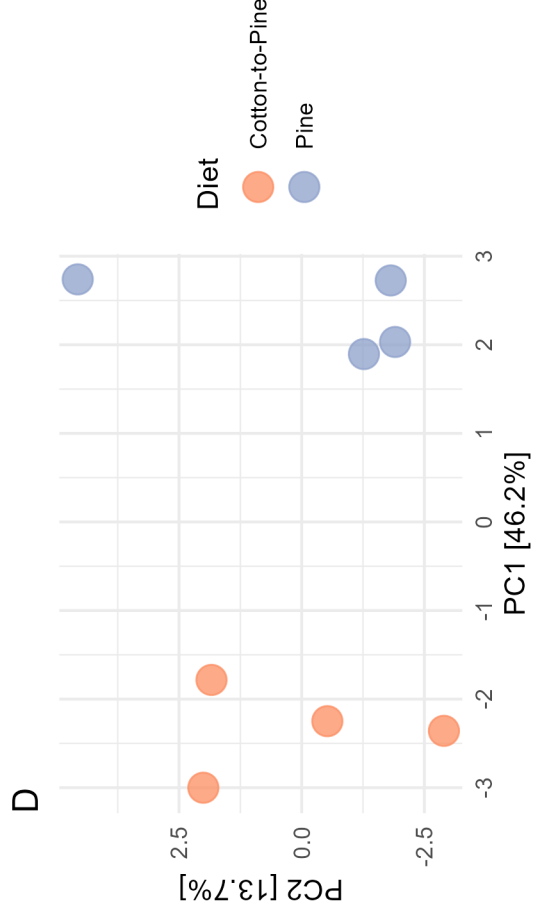

Supplement: Supplemental Information 2 — A cotton and cotton to pine, B pine and pine to cotton, C cotton and pine to cotton, and D pine and cotton to pine. Each PCA was performed on the aitchison distances of a centred log ratio transformed ASVs data set. A PERMANOVA using each of the two diets was also: A, R2 = 0.41, F-statistic = 4.21, p-value = 0.031; B, R2 = 0.31, F-statistic = 2.7, p-value = 0.031; C, R2 = 0.15, F-statistic = 1.1, p-value = 0.381; D, R2 = 0.45, F-statistic = 4.93, p-value = 0.035. [file peerj-12-17597-s002.pdf]

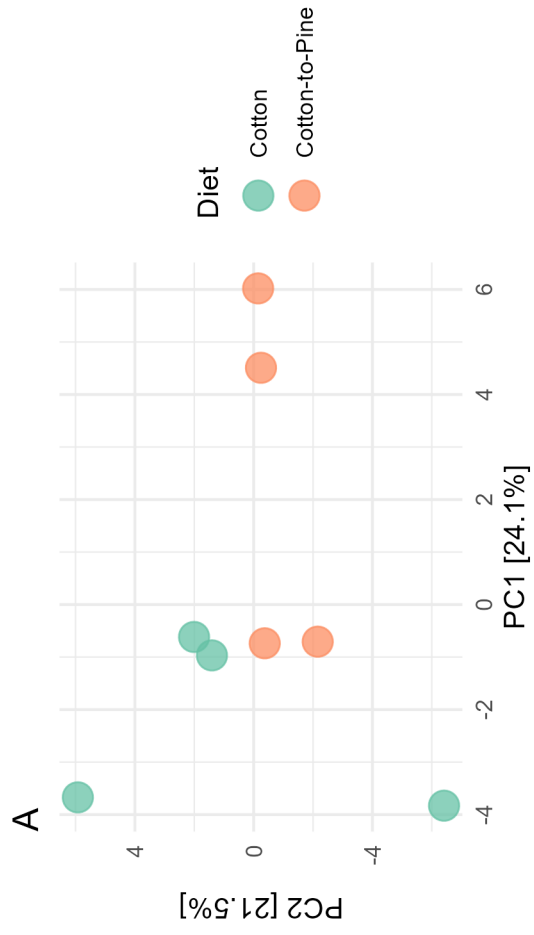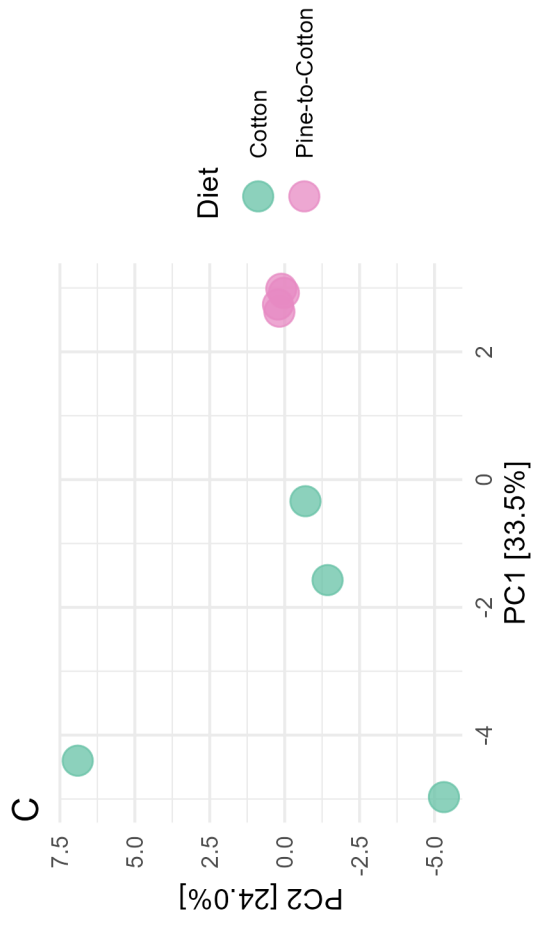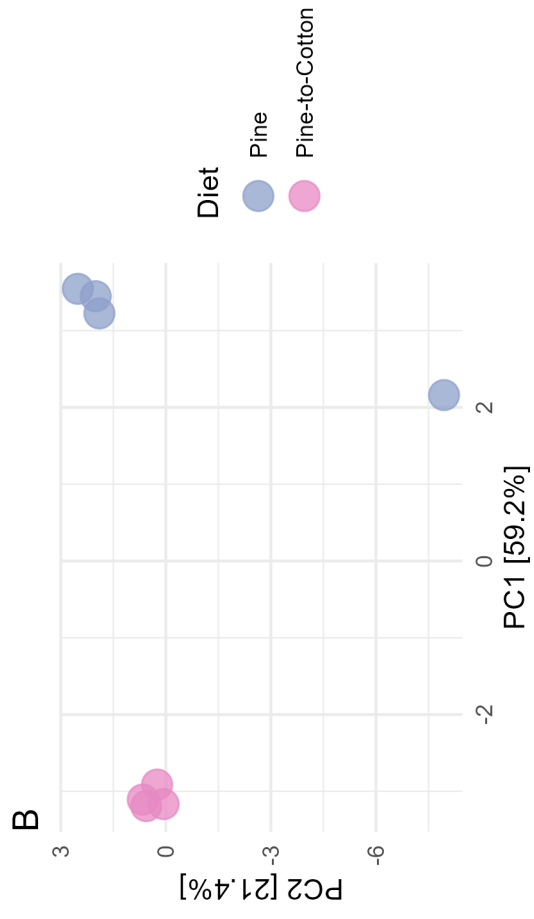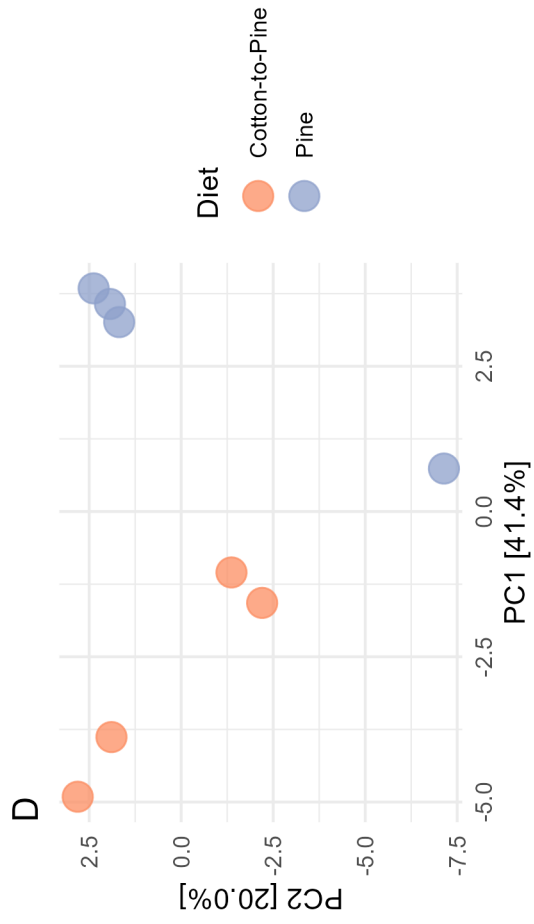

Supplement: Supplemental Information 3 — A cotton and cotton to pine, B pine and pine to cotton, C cotton and pine to cotton, and D pine and cotton to pine. Each PCA was performed on the aitchison distances of a centred log ratio transformed ASVs data set. A PERMANOVA using each of the two diets was also: A, R2 = 0.19, F-statistic = 1.39, p-value = 0.056; B, R2 = 0.59, F-statistic = 8.48, p-value = 0.031; C, R2 = 0.31, F-statistic = 2.63, p-value = 0.035; D, R2 = 0.36, F-statistic = 3.42, p-value = 0.030 [file peerj-12-17597-s003.pdf]
